# Serotonin in Health and Disease

**DOI:** 10.3390/ijms21103500

**Published:** 2020-05-15

**Authors:** Philippe De Deurwaerdère, Giuseppe Di Giovanni

**Affiliations:** 1 Centre National de la Recherche Scientifique, UMR CNRS 5287, CEDEX F-33000 Bordeaux, France; 2 Laboratory of Neurophysiology, Department of Physiology and Biochemistry, Faculty of Medicine and Surgery, University of Malta, MSD2080 Msida, Malta; 3 School of Biosciences, Neuroscience Division, Cardiff University, Cardiff CF10 3AX, UK

## Abstract

The International Journal of Molecular Sciences Special Issue “Serotonin in health and diseases” covers several aspects of the multiple and still mysterious functions of serotonin (5-hydroxytryptamine; 5-HT). 5-HT is neurotransmitter acting in the central nervous system (CNS), blood factor, and neurohormone controlling the function of several peripheral organs. Beyond its widespread implication in physiology, the 5-HT system is involved in numerous diseases of the CNS (e.g., depression, anxiety, schizophrenia, obsessive-compulsive disorders, addiction, Parkinson’s disease) and peripheral organs (e.g., gastrointestinal disorders, cardiac arrhythmia, hypertension). The Special Issue includes 14 articles dealing with molecular and cellular effects of 5-HT in periphery and CNS, from functional aspects in lower animals to clinical practices. Beyond physiology, the Special Issue also covers the influence of 5-HT and its receptors in the mechanism of action of psychoactive molecules including antipsychotics, antidepressants, and drug of abuse. The recent progress made on the function and dysfunction of the 5-HT system will certainly increase the understanding of the widespread role of 5-HT ultimately leading to better apprehend its targeting in human diseases.

The International Journal of Molecular Sciences (IJMS) Special Issue “Serotonin in health and diseases” covers several aspects of the multiple and still mysterious functions of serotonin (5-hydroxytryptamine; 5-HT). 5-HT is neurotransmitter acting in the central nervous system (CNS), blood factor, and neurohormone controlling the function of several peripheral organs. Beyond its widespread implication in physiology, the 5-HT system is involved in numerous diseases of the CNS (e.g., depression, anxiety, schizophrenia, obsessive-compulsive disorders, addiction, Parkinson’s disease) and peripheral organs (e.g., gastrointestinal disorders, cardiac arrhythmia, hypertension). Numerous drugs targeting the 5-HT system are currently used in the clinic for different purposes. The recent progress made on the function and dysfunction of the 5-HT system will certainly increase their number.

An interesting emphasis on the early 5-HT mechanisms occurring in the development is reported in the Special Issue. Notably, Nikishin et al. report the expression and functional activity of the 5-HT transporter (SERT) and the enzyme for the synthesis of 5-HT, aromatic l-amino acid decarboxylase (DDC) and tryptophan hydroxylase (TPH) in mouse ovary [1]. While 5-HT is present in oocytes at stages as early as 14 days, the enzymes DDC and TPH are poorly active, merely accounting for the accumulation of 5-HT in these cells. The authors propose that the presence of 5-HT in oocytes at early stages would be mainly due to the reuptake by the SERT among the functions of 5-HT occurring at embryonic stages and are the maturations of neurobiological networks. Martin et al. show that 5-HT, present at the lumbar level of mouse spinal cord, indirectly regulates via 5-HT receptors the chloride homeostasis of motoneurons by modulating the function of the chloride transporter KCC2 [2]. The temporal window is extremely precise, occurring at E17.5 of the development and impairment of 5-HT function could account for the abnormal maturation of motoneurons in SODG93A mice, a model of amyotrophic lateral sclerosis. Indeed, the authors show that 5-HT content and fibers are reduced at the lumbar level at E17.5 in SOD1G93A compared to their wildtype littermate.

The mechanisms triggered by 5-HT at the cellular level are complex, as reported by Tada et al. for the responses of the porcine intramuscular mature adipocytes [3]. The authors propose a transcriptomic study on the consequences of differentiation of intramuscular preadipocytes or the responses of mature adipocytes to 5-HT and the factor TNFα. 5-HT application changed the transcriptome as the two other conditions. Nonetheless, at variance with the other conditions, transcriptomic changes induced by 5-HT concerned genes involved in the significant enrichment of GPCR ligand binding, cell chemotaxis, regulation of lipid metabolism and transport pathways to cite a few. It recalls that 5-HT can be seen as an orchestrator of metabolism and cell interaction. Modification of 5-HT function should draw specific attention as indicated by Slawomir Gonkowski [4]. In his study performed in the porcine intestine, he found that the exposition of pigs to Bisphenol A, a substance used in the production of plastics, enhances the number of 5-HT cells along with the mucosal layer of the duodenum, jejunum, and ileum.

The CNS federates the majority of the studies on the 5-HT systems but their function is still enigmatic. Bacque-Cazenave et al. propose an interesting review of the role of 5-HT in animal cognition and behavior [5]. Despite the distinct organization of the 5-HT system in lower animals compared to vertebrates, its function is quite well conserved across the animal kingdom when looking at functions as diverse as locomotion, feeding, sleep and circadian rhythms, anxiety, aggressiveness and social status, learning and memory, and mood. 5-HT would be an essential system for regulating adaptive behaviors in the phylum. Deryabina et al. report a study in snails focusing on the mechanisms of memory reconsolidation through the angle of 5-HT [6]. They found that the TPH inhibitor para-chlorophenylalanine can impair the contextual memory reconsolidation. The role of 5-HT in learning and memory is of importance in the context of Alzheimer’s disease and several 5-HT receptors, notably 5-HT6 receptors have been proposed to be targeted in the disease. Latacz et al. report a new family of triazine-piperazine derivatives targeting 5-HT_6_ receptors, notably 4-((2-isopropyl-5-methylphenoxy)methyl)-6-(4-methylpiperazin-1-yl)-1,3,5-triazin-2-amine [7]. They describe the pharmacological profile, the safety, and the positive cognitive and anxiolytic properties of their compounds. Another function regulated by 5-HT is reward. Using two constructs to optogenetically stimulating the 5-HT neurons of the dorsal raphe nucleus (DRN), Nagai et al. report that the stimulation of DRN 5-HT fibers increased nose-poke self-stimulation test and the conditioned place preference [8]. They conclude that 5-HT neurons from the DRN, perhaps by the interaction they establish with dopaminergic neurons of the ventral tegmental area, are a key component in the balance between reward and aversion.

The interaction of 5-HT with dopaminergic neurons is, in fact, essential in the study of the mechanisms of numerous neuroactive drugs [9] including the drug of abuse or antipsychotics. Grubor et al. have studied the responses of schizophrenic patients to the classical neuroleptic haloperidol with regards to *HTR1A*, *HTR1B*, *HTR2A*, *HTR2C*, and *HTR6* gene polymorphisms and extrapyramidal side effects [10]. Interestingly, rather than the expected influence of HT2A or HT2C gene polymorphisms based on the knowledge of the 5-HT/dopamine interaction, the authors report a clear association of the *HT1B* gene polymorphism with one of the extrapyramidal side effects of classical antipsychotics, namely akathisia. It is an interesting discovery because akathisia is still a concern with newer antipsychotics. The influence of the 5-HT system on dopaminergic responses likely occurs in several brain regions. Bombardi et al. show that acute and chronic treatments with nicotine in rats differently alter the electrophysiological responses of the lateral habenula (LHb) neurons to the peripheral administration of the 5-HT_2A_ receptor agonist TCB-2 [11]. By looking at the immunostaining of 5-HT_2A_ receptors in numerous brain regions including the hippocampus, LHb, DRN, medial prefrontal cortex to cite a few, they found specific, regional changes of the 5-HT_2A_ receptor distribution that differ between acute and chronic nicotine treatment that might be involved in its addictive effect. Di Giovanni and colleagues’ findings [11] further suggest that 5-HT_2A_ receptor agonists sustain nicotine addictive properties, while antagonists may have a role in the development of new treatment for nicotine dependence in humans.

The idea that the interaction between the 5-HT and dopaminergic systems is broader than previously thought is also supported by the data reported by Whitestone et al. [12]. They studied the neurochemical effect of the 5-HT_2C_ receptor agonist WAY-163909 on the metabolism of dopamine and 5-HT across 29 brain regions. Despite the known and numerous behavioral effects triggered by this compound, they found very few quantitative changes in the dopaminergic and 5-HT metabolisms. Conversely, they report a widespread change in the relationships between the metabolisms across the brain regions using systematic correlative analyses of these metabolisms.

Summing up, 5-HT plays a role in organizing the responses of the developing and mature tissues and the function of the 5-HT system can be modified by environmental factors and drugs. While the environmental toxins can have dramatic effects on 5-HT mechanisms, the SERT inhibitors (SRIs) are classically given for the treatment of depression and mood disorders. The article of Okada et al. is an interesting neurochemical contribution to the mechanism of action of two antidepressant drugs having a distinct pharmacological profile, namely vortioxetine and escitalopram [13]. Measuring 5-HT extracellular levels using intracerebral microdialysis, they found that both vortioxetine which has a complex pharmacological in addition to being an SRI, and escitalopram, which is a selective SRI, lead to the desensitization of 5-HT_1A_ receptors in the prefrontal cortex and the DRN, upon subchronic administration. Their mechanisms differed with regards to the cortical 5-HT_3_ receptors, desensitized in the case of escitalopram but not vortioxetine. In any case, the article by Okada et al. nicely recalls that it is upon their chronic administration that SRIs boost 5-HT transmission. While this mechanism is likely at the origin of their benefit, the increase in 5-HT extracellular levels produced by these compounds could have undesirable effects on the development of the fetus when they are given to pregnant mothers. It can cause transient developmental retardation in children by acting on different systems regulated by 5-HT, as summarized by Ornoy and Koren [14]. The consequences are not always clear, and the authors balance the risk of continuing SRIs with the risk of discontinuing SRIs in depressed mothers. In the general population, the use of medicines targeting the 5-HT system, notably the antidepressant drugs, can lead to one devastating clinical outcome termed the 5-HT syndrome [15]. Francescangeli et al. describe the 5-HT syndrome and its clinical manifestation. They further dissociate through a molecular analysis in the 5-HT syndrome with similar conditions.

The clinical and preclinical studies collected in this Special Issue confirm that the 5-HT system is an important modulator of central and peripheral functions that reacts to drugs and environmental factors. It is still a target of choice for numerous diseases and additional contributions will surely enlarge our knowledge of this chemical factor likely serving as adaptive glands to adjust the responses of the organisms.
